# Compensatory elevation of voluntary activity in mouse mutants with impaired mitochondrial energy metabolism

**DOI:** 10.14814/phy2.12214

**Published:** 2014-11-20

**Authors:** Jérôme Lapointe, Bryan G. Hughes, Eve Bigras, Siegfried Hekimi

**Affiliations:** Department of Biology, McGill University, Montréal, Quebec, Canada

**Keywords:** Behavior, energy metabolism and oxidative stress, *Mclk1* RISP *Sod2*, mitochondria

## Abstract

Mitochondria play a crucial role in determining whole‐body metabolism and exercise capacity. Genetic mouse models of mild mitochondrial dysfunction provide an opportunity to understand how mitochondrial function affects these parameters. MCLK1 (a.k.a. Coq7) is an enzyme implicated in the biosynthesis of ubiquinone (UQ; Coenzyme Q). Low levels of MCLK1 in *Mclk1*^+/−^ heterozygous mutants lead to abnormal sub‐mitochondrial distribution of UQ, impaired mitochondrial function, elevated mitochondrial oxidative stress, and increased lifespan. Here, we report that young *Mclk1*^+/−^ males, but not females, show a significant decrease in whole‐body metabolic rate as measured by indirect calorimetry. Such a sex‐specific effect of mitochondrial dysfunction on energy metabolism has also been reported for heterozygous mice carrying a mutation for the gene encoding the “Rieske” protein of mitochondrial complex III (*RISP*^+/*P224S*^). We find that both *Mclk1*^+/−^ and *RISP*^+/*P224S*^ males are capable of restoring their defective metabolic rates by making significantly more voluntary use of a running wheel compared to wild type. However, this increase in voluntary activity does not reflect their exercise capacity, which we found to be impaired as revealed by a shorter treadmill distance run before exhaustion. In contrast to what is observed in *Mclk1*^+/−^ and *RISP*^+/*P224S*^ mutants, *Sod2*^+/−^ mice with elevated oxidative stress and major mitochondrial dysfunction did not increase voluntary activity. Our study reveals a sex‐specific effect on how impaired mitochondrial function impacts whole‐body energy metabolism and locomotory behavior, and contributes to the understanding of the metabolic and behavioral consequences of mitochondrial disorders.

## Abstract (precis)

Mitochondria play a crucial role in determining whole‐body metabolism, lifespan and exercise capacity. This study reports sex‐specific effects of mitochondrial dysfunction, resulting in increased spontaneous activity in response to impaired metabolic rates. These findings contribute to the understanding of the metabolic and behavioral consequences of mitochondrial disorders.

## Introduction

MCLK1 is a mitochondrial enzyme that catalyzes the hydroxylation of 5‐demethoxyubiquinone (DMQ) to form 5‐hydroxyubiquinone, a crucial step in the ubiquinone (UQ) biosynthesis pathway (Dallner and Sindelar 2000; Stepanyan et al. 2006). Ubiquinone, also known as coenzyme Q (CoQ), is a redox‐active molecule localized in cellular membranes and characterized by a benzoquinone ring coupled to a lipophilic side‐chain with a species‐specific number of isoprene subunits (Wang and Hekimi 2013). The principal functions attributed to UQ are as an electron carrier in the mitochondrial electron transport chain (ETC) and, in its reduced form, as an antioxidant that protects membranes against the toxic actions of reactive oxygen species (ROS; Bentinger et al. 2010). Previous work from our laboratory has revealed that reduced levels of MCLK1 are associated with increased lifespan in both *Caenorhabditis elegans* and mouse mutants (Ewbank et al. 1997; Liu et al. 2005). Indeed, while the complete inactivation of *Mclk1* results in embryonic lethality in mice (Levavasseur et al. 2001), *Mclk1* heterozygous mutants live significantly longer and accumulate biomarkers of aging more slowly than their wild‐type siblings (Liu et al. 2005; Lapointe et al. 2009). This increase in longevity has been linked to impaired mitochondrial energy metabolism in young *Mclk1*^+/−^ mice (Lapointe and Hekimi 2008). Reduced oxygen consumption capacity was observed with several metabolic substrates in isolated mitochondria from different tissues and a correlation with both electron transport rate and ATP production was established. Unexpectedly for a long‐lived model, significant mitochondrial oxidative stress was also observed in young *Mclk1*^+/−^ mice (Lapointe and Hekimi 2008, 2010). A recent analysis has revealed that these mitochondrial defects are due to an abnormal distribution of UQ in mitochondrial membranes (Lapointe et al. 2012). UQ levels were found to be lower than normal in the inner mitochondrial membrane while they were higher in the outer membrane.

Partial inhibition of another constituent of the mitochondrial ETC, the “Rieske” iron‐sulfur protein (RISP, a.k.a. the ubiquinol‐cytochrome c reductase rieske iron‐sulfur polypeptide 1, UQCRFS1), also affects mortality variables and mitochondrial function in mice (Hughes and Hekimi 2011). RISP is one of the core components of mitochondrial complex III, which accepts electron from ubiquinol (Iwata et al. 1998). In the *isp‐1* gene, the *C. elegans* homolog of *Risp*, a single base substitution that changes a conserved proline into a serine dramatically extends lifespan of the mutants (Feng et al. 2001). A knock‐in mouse strain that carries the same amino acid substitution (P224S) in the functional domain was generated and found to be embryonic lethal when homozygous for the mutation (Hughes and Hekimi 2011). Heterozygous mice (*RISP*^+/*P224S*^) had partially impaired complex III activity and decreased mitochondrial oxygen consumption. Analysis of Gompertz parameters showed that *Risp* heterozygosity decreased the rate of increase in mortality with age but increased the intrinsic vulnerability to death in both sexes.

Interestingly, evaluation of whole‐body energy metabolism by indirect calorimetry indicated that the mitochondrial dysfunction is sufficient to impair metabolic rate in heterozygous male *RISP*^+/*P224S*^ mutants, but not in females (Hughes and Hekimi 2011). It is well accepted that at least 90% of the resting metabolic rate is attributable to mitochondrial respiration but the study of *RISP*^+/*P224S*^ mutants was one of the very few demonstrations that directly relates a defined mitochondrial defect to a measurable effect at the level the whole organism not complicated by severe disease phenotypes (Rolfe and Brown 1997). Several studies indicated that mitochondrial energy production is also perturbed in mitochondria isolated from mice heterozygous for the major mitochondrial enzymatic defense against superoxide, the manganese superoxide dismutase (*MnSOD* or *Sod2*). Like *Mclk1*^+/−^ mutants, *Sod2*^+/−^ mutants sustain significant mitochondrial oxidative stress (Williams et al. 1998; Melov et al. 1999). Assessment of the in vivo consequences on metabolic rate revealed that both oxygen consumption and carbon dioxide production were altered in *Sod2*^+/−^ mice (Kinugawa 2005). Moreover, exercise capacity is limited in these mutants as shown by treadmill endurance tests (Lustgarten et al. 2009).

To further study how mitochondrial dysfunction resulting from genetic mutations affects metabolic rate and physical performance, we have evaluated a variety of physiological parameters in *Mclk1*^+/−^, *RISP*^+/*P224S*^*,* and *Sod2*^+/−^ mutants. We found that whole‐body metabolic rate is affected in *Mclk1*^+/−^ males but not in females. We further observed a significant increase in voluntary wheel‐running activity in both sexes. As a result, whole‐body energy metabolism tended to be higher and respiratory exchange ratio significantly lower (toward fat oxidation) in *Mclk1*^+/−^ males in the presence of a running wheel. Strikingly, similar behavioral and physiological changes were observed for the *RISP*^+/*P224S*^ males which also restore their low resting metabolism by being more active on the running wheel. On the other hand, evaluation of the exercise capacity of the *Mclk1*^+/−^ males indicated that they performed worse than controls when subjected to forced activity on a treadmill. Furthermore, we report that voluntary activity is greater in *Sod2*^+/−^ *Mclk1*^+/−^ double mutants than in *Sod2*^+/−^ mutants, a partial rescue similar to what we observed previously for mitochondrial function and oxidative stress (Lapointe et al. 2009). The present study highlights that independent genetic mutations affecting different parts of the mitochondrial electron transport chain can result in similar whole‐body metabolic and behavioral changes. It further reveals that impaired mitochondrial energy production affecting baseline metabolic rates can be found to be compensated for by an increase in the level of voluntary activity in mitochondrial mutants. Lastly our study provides additional evidence that mitochondrial oxidative stress decreases exercise capacity.

## Methods

### Animals

All the animals were housed in a pathogen‐free facility at McGill University, 2–5 per cage, and were given a standard rodent diet and water ad libitum. Generation and breeding strategies for the *Mclk1*^*+/−*^*, RISP*^*+/P224S*^*,* and *Sod2*^+/−^ mice in their respective genetic background have been previously described (Huang et al. 2001; Levavasseur et al. 2001; Lapointe et al. 2009; Hughes and Hekimi 2011). All studies were approved by the McGill Faculty of Science Animal Care Committee and conducted according to the guidelines of the Canadian Council on Animal Care.

### Indirect calorimetry

Whole‐body energy metabolism was measured with an indirect calorimetry system including eight individual chambers (Oxymax; Columbus Instruments, Columbus, OH). Mice were weighed prior the experiment and placed in the apparatus for 24 h to allow them to acclimate before measurements were started. Oxygen consumption and carbon dioxide production was then recorded continuously during a 12 h light and 12 h dark cycle for 24 h with ad libitum feeding. These measurements were then used to calculate the respiratory exchange ratio and heat production. Metabolic rates were normalized to total body weight apart for those related to the *RISP*^*+/P224S*^ and their control siblings which were instead normalized to the combined weight of the liver, brain, heart, and kidneys. This latter method of normalization has also been shown to accurately account for the rate of energy consumption (Greenberg and Boozer 2000). Results were averaged over 2 h intervals in order to smooth out the substantial point‐to‐point variation.

### Voluntary activity

Running wheels were added in each of the eight cages of the indirect calorimetry system (Oxymax; Columbus Instruments) and individually housed mice were then allowed to use them voluntarily. Mice were weighed prior to the experiment and placed in the apparatus for 24 h to allow them to acclimate before measurements were started. Wheel turns, oxygen consumption and carbon dioxide production were recorded continuously during a 12 h light and 12 h dark cycle with ad libitum feeding for either 48 h for *RISP*^*+/P224S*^ and their control siblings or 60 h for all the other genotypes tested. Respiratory exchange ratio and heat production were calculated from these measurements. Results were again averaged over 2 h intervals.

### Treadmill exercise capacity test

Mice were run on a treadmill (Columbus Instruments) set at a 10% incline to evaluate exercise capacity. To acclimatize mice to this form of exercise, mice were run at 6 m/min for 5 min. Exercise capacity was then determined by graded increases in treadmill speed (6, 10, and 15 m/min for 3 min at each speed) followed by 2 m/min increase every 3 min to exhaustion. Oxygen and carbon dioxide gas fractions were continuously monitored at both the inlet and output ports of the metabolic chamber. Exhaustion was determined by a failure to engage the treadmill in the presence of a mild shock (10 sec on the shocker plate).

### Measurement of biochemical metabolites

The mouse tail was nicked with a needle, and the tail vein was massaged to obtain an appropriate volume of blood for glucose and lactate measurements. Blood glucose was measured using an Accu‐Check Aviva blood glucose meter (Roche, Mannheim, Germany). Blood lactate was measured using the Lactate Pro meter (Arkray Inc., Kyoto, Japan). Glucose and lactate levels were measured before and after the treadmill exercise capacity test.

### Statistics

Group data were presented as mean values ± SEM. Quantitative data were analyzed by GraphPad Prism Version 5.00 for Windows (GraphPad Software Inc., San Diego, CA). Comparisons between two groups were performed using an unpaired two‐tailed Student's *t‐*test. For multiple comparisons, one‐way analysis of variance (ANOVA) followed by Bonferroni's post hoc analysis was performed. Repeated‐measures two‐way ANOVA was used to determine statistical significance for indirect calorimetry. For all analyses, a value of *P* < 0.05 was considered significant.

## Results

### A sex‐specific decrease in metabolic rate in Mclk1^+/−^ mice

**Figure 1.**
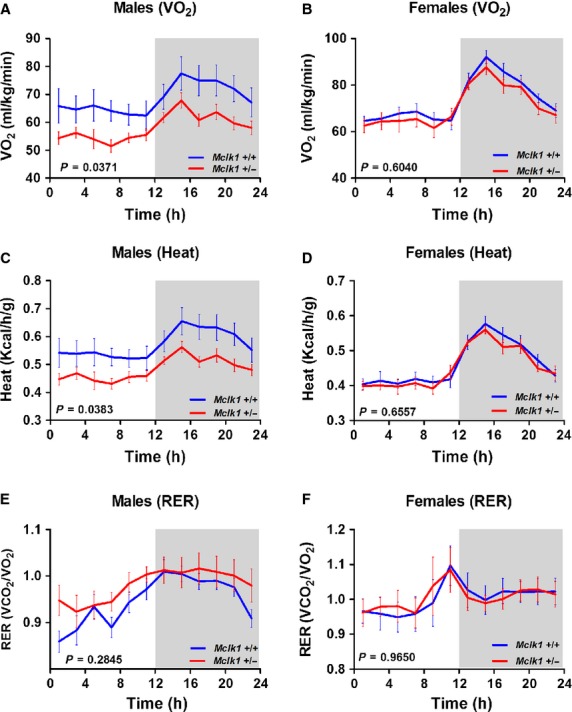
Sex‐specific decreased in whole‐body metabolism in *Mclk1*^+/−^ mice. Resting metabolic parameters were continuously assessed by indirect calorimetry performed over a 24 h period with a 12 h light and 12 h dark cycle in 3‐month‐old *Mclk1*^+/+^ and *Mclk1*^+/−^ mice (*n* = 10–12). Significant changes in oxygen consumption rate (A) and heat production (C) were observed between genotypes for males. In contrast, oxygen consumption (B), heat production, (D) and respiratory exchange ratio (E,F) were similar between *Mclk1*^+/+^ and *Mclk1*^+/−^ females throughout the 24 h period. The shaded areas demarcate the dark phases (from 7:00 pm to 7:00 am). Time “0” is 7:00 am. All points represent means ± SEM averaged over 2 h intervals. A value of *P* < 0.05 for the genotype effect was considered significant.

All mice were weighed prior to the experiments and no differences were observed between heterozygotes and controls (data not shown). We have previously reported that there is also no differences in body composition (Liu et al. 2005; Lapointe and Hekimi 2008). Similarly, an evaluation of both body weight and food intake at 3, 12, and 23 months of age did not show significant differences between genotypes, with the exception of 23‐month‐old *Mclk1*^+/−^ females that were found to be slightly heavier than their wild‐type siblings (data not shown). Whole‐body metabolic parameters were assessed by indirect calorimetry in 3‐month‐old male and female *Mclk1*^+/−^ mice in the Balb/c genetic background (Fig. 1). Analysis of oxygen consumption (VO_2_) showed the expected diurnal pattern characterized by an increased VO_2_ during the dark period, when mice are more active, compared to the light period (Fig. 1A and B). Throughout the 24 h period, the *Mclk1*^+/−^ male mice showed decreased VO_2_ compared to controls whereas the slight reduction observed for *Mclk1*^+/−^ females did not reach significance (Fig. 1A and B). Similar results were obtained for heat production, which is a calculated measure of metabolic rate. We observed the predicted diurnal rhythm with increased heat production during the night and found that, in contrast to the females, the *Mclk1*^+/−^ males produced less heat than controls (Fig. 1C and D). We also measured the respiratory exchange ratio (RER) which correspond to the volume of carbon dioxide produced over a given time (VCO_2_) divided by the oxygen that was simultaneously consumed (RER = VCO_2_/VO_2_) and indicates which substrates are preferably oxidized. A ratio near 1 indicates lipid oxidation, while a value of 0.7 is an indication of carbohydrate oxidation. We observed a trend toward fat oxidation as the experimental period progressed from day to night and no statistically significant differences in RER ratio could be observed between *Mclk1*^+/+^ and *Mclk1*^+/−^ mice for both sexes (Fig. 1E and F).

### Mclk1^+/−^ mice display increased voluntary activity and enhanced metabolic rate in the presence of a running wheel

**Figure 2.**
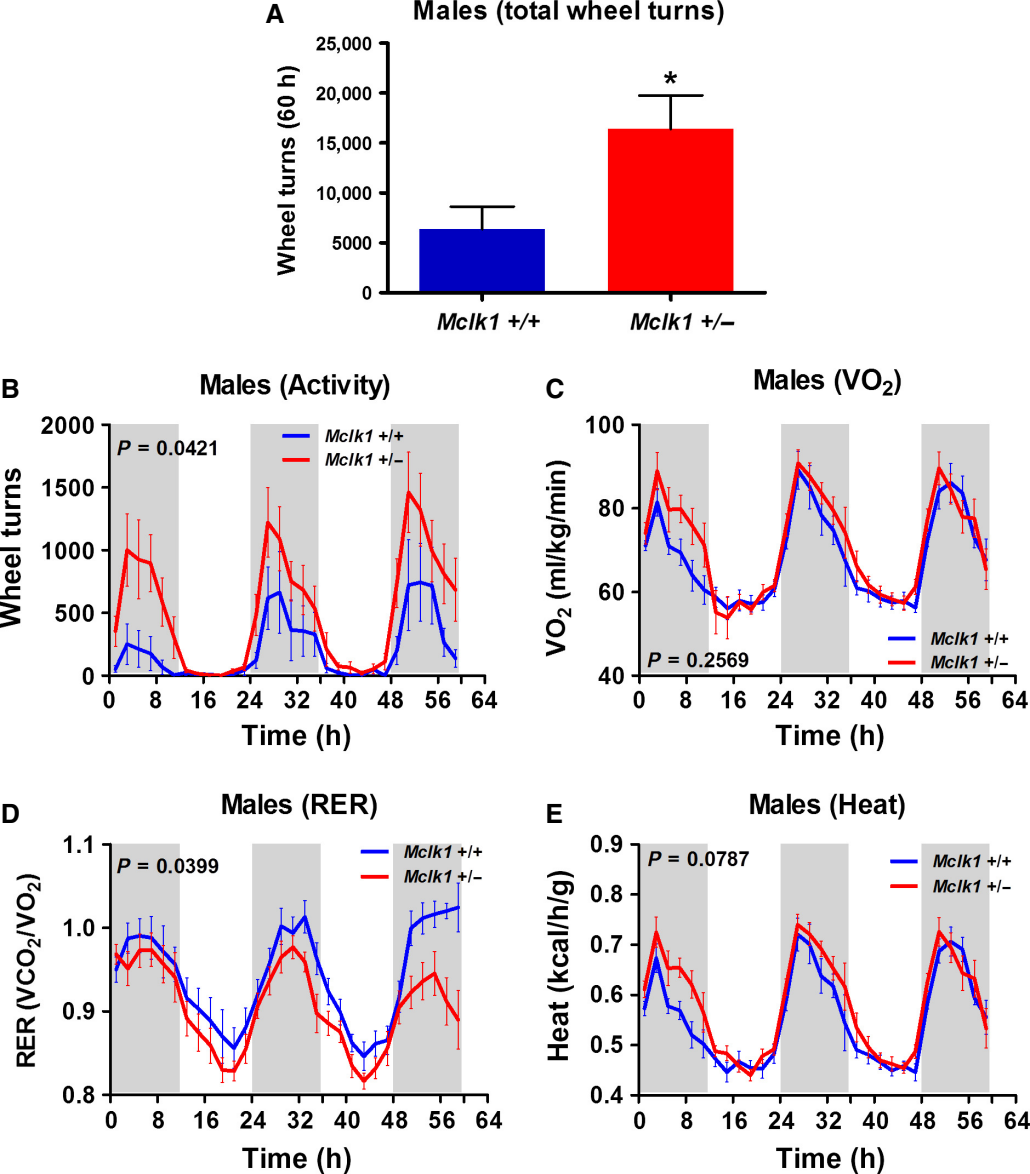
Increased voluntary running wheel activity and metabolic rate in *Mclk1*^+/−^ males. Total number of wheel turns accomplished by 3‐month‐old *Mclk1*^+/+^ and *Mclk1*^+/−^ mice (*n* = 10–12) during a 60 h period with a 12 h light and 12 h dark cycle. (A). Bars represent means ± SEM and a value of *P* < 0.05 was considered significant. Voluntary activity on running wheels was continuously recorded throughout the 60 h period (B). Metabolic rate parameters were also assessed by indirect calorimetry. The shaded areas demarcate the dark phases (from 7:00 pm to 7:00 am). Time “0” is 7:00 am. Changes in oxygen consumption rate (C), respiratory exchange ratio (D) and heat production (E) and were reported. All points represent means ± SEM over 2 h intervals. A value of *P* < 0.05 for the genotype effect was considered significant.

**Figure 3.**
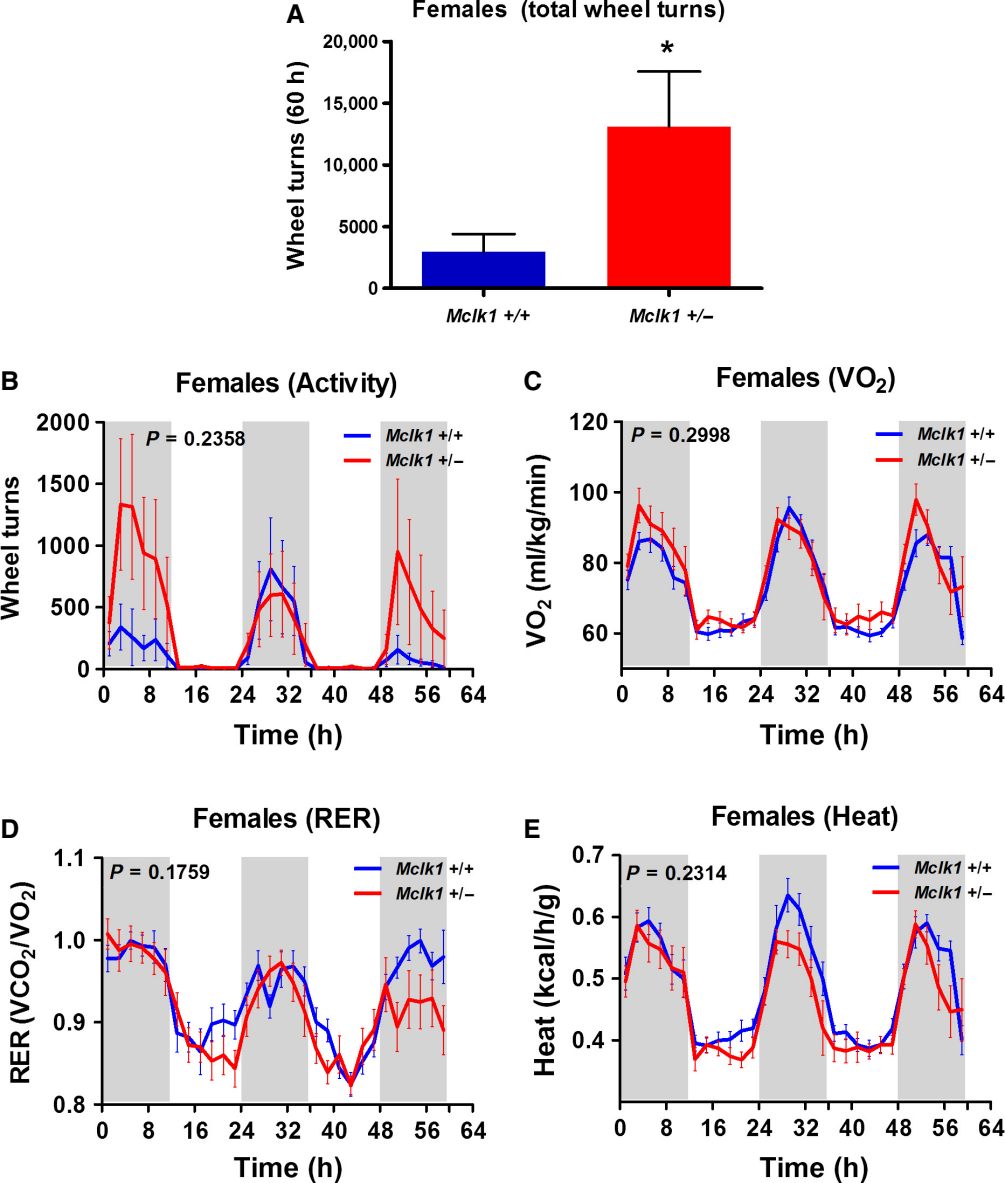
Voluntary running wheel activity and metabolic rate in *Mclk1*^+/+^ and *Mclk1*^+/−^ females. Total number of wheel turns accomplished by 3‐month‐old *Mclk1*^+/+^ and *Mclk1*^+/−^ females (*n* = 10–12) during a 60 h period with a 12 h light and 12 h dark cycle. (A). Bars represent means ± SEM and a value of *P* < 0.05 was considered significant. Voluntary activity on a running wheel was continuously recorded throughout the 60 h period (B). Metabolic rate parameters were also assessed by indirect calorimetry. The shaded areas demarcate the dark phases (from 7:00 pm to 7:00 am). Time “0” is 7:00 am. Changes in oxygen consumption rate (C), respiratory exchange ratio (D) and heat production (E). All points represent means ± SEM over 2 h intervals. A value of *P* < 0.05 for the genotype effect was considered significant.

We tested the effects of impaired metabolism on spontaneous activity and vice versa by giving animals 24‐h access to a running wheel (Figs. 2, 3). After a period of adaptation, the total number of wheel turns was calculated for the subsequent 60 h (with 12 h dark/light intervals). As expected, mice of all groups were much more active on the running wheels during the dark periods (Figs. 2B, 3B). However, the total number of wheel turns produced throughout the 60 h was much higher than wild‐type controls for *Mclk1*^+/−^ mutants of both sexes (Figs. 2A, 3A). A threefold statistically significant increase in voluntary activity was observed for the heterozygotes. This increase was clearly seen throughout the experimental period for the males but, for unknown reasons, was observed only in the first and last dark periods for the females (Figs. 2B, 3B). The presence of running wheels in the metabolic cages rescued the decreased VO_2_ and heat production observed without running wheels in *Mclk1*^+/−^ males (Fig. 2C and E; compare to Fig. 1A and C). In addition, the respiratory exchange ratio was found to be significantly lower in *Mclk1*^+/−^ males (Fig. 2D). Metabolic rates were not further increased for *Mclk1*^+/−^ females in presence of running wheels (Fig. 3C–E; compare to Fig. 1).

### The voluntary activity phenotype of Sod2^+/−^ mutants is rescued in Sod2^+/−^ Mclk1^+/−^ double mutants

**Figure 4.**
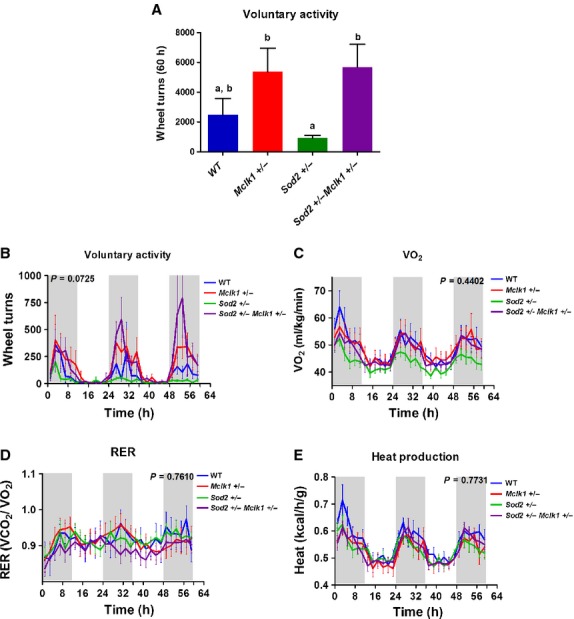
Voluntary activity in 15‐month‐old *Mclk1*^+/−^, *Sod2*^+/−^, and *Mclk1*^+/−^ *Sod2*^+/−^ mutants. Assessment of whole‐body metabolic rate and voluntary activity in 15‐month‐old wild‐type, *Mclk1*^+/−^, *Sod2*^+/−^, and *Sod2*^+/−^ *Mclk1*^+/−^males on the DBA/2J/B6 F1 background. Total number of wheel turns accomplished during a 60 h period (A). Bars represent means ± SEM. Bars with different letters (a, b) differ from each other while bars with a letter in common are not statistically different (*P* < 0.05). Continuous recording of voluntary activity on running wheel through a 60 h period with a 12 h light and 12 h dark cycle. (B). Metabolic rate parameters were also assessed by indirect calorimetry for the entire experimental period. The shaded areas demarcate the dark phases (from 7:00 pm to 7:00 am). Time “0” is 7:00 am. Changes in oxygen consumption (C), respiratory exchange ratio (D) and heat production (E). All points represent means ± SEM over 2 h intervals. A value of *P* < 0.05 for the genotype effect was considered significant.

*Sod2*^+/−^ mice sustain mitochondrial oxidative stress that affects their metabolic rate and exercise capacity (Williams et al. 1998; Kinugawa 2005). We have previously shown that at 15 months of age these features are dramatically alleviated in double heterozygous *Sod2*^+/−^ *Mclk1*^+/−^ animals (Lapointe et al. 2009). The metabolic rate of such 15‐month‐old male mice of similar body weight was first analyzed without access to running wheels and no significant differences between genotypes were observed for VO_2_, RER or heat production (data not shown). This contrasts with our findings with 3‐month‐old *Mclk1*^+/−^ males. However, we have previously shown that most phenotypes of *Mclk1*^+/−^ mice evolve toward control values with aging (Lapointe et al. 2009). The 15‐month‐old male mice were then subjected to voluntary activity analysis for a 60 h period by allowing them continuous access to a running wheel. As expected, based on previous research, *Sod2*^+/−^ animals were not very active, although the difference with the wild‐type controls did not reach significance (Fig. 4A). However, the total number of wheel turns accumulated by the double heterozygotes was about fivefold greater than that recorded for *Sod2*^+/−^ mutants (Fig. 4A). This difference between the genotypes tended to be consistently apparent throughout the 60 h period (Fig. 4B). Analysis of the VO_2_, RER and heat production did not reveal any effect of genotype throughout the experimental period (Fig. 4C–E).

### Decreased exercise capacity of Mclk1^+/−^ mutants

**Figure 5.**
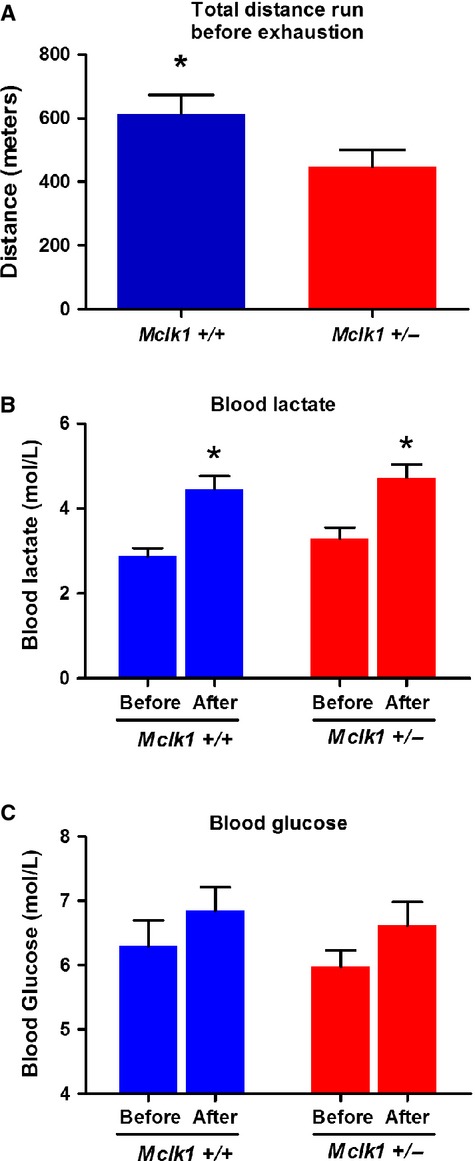
Decreased exercise capacity in *Mclk1*^+/−^ male mice. Assessment of forced exercise capacity was performed with 3‐month‐old *Mclk1*^+/+^ and *Mclk1*^+/−^ mice (*n* = 18) on the Balb/C background. Total distance run on the treadmill by both genotypes (A). Blood levels of lactate (B) and glucose (C) from the tail vein before and after the endurance test. Bars represent means ± SEM and a value of *P* < 0.05 was considered significant.

The increased voluntary activity observed in *Mclk1*^+/−^ mutants, which may be a behavioral adaptation to their intrinsic mitochondrial dysfunction, did not provide any information about their exercise capacity. For this we carried out a physical endurance task on a treadmill (see Methods). We scored the mean total distance that the mice were able to run before exhaustion in 3‐month‐old *Mclk1*^+/−^ males and sibling controls. The mutants exhibited significantly impaired exercise capacity (Fig. 5A). As expected, the treadmill endurance test significantly increased the animals' blood lactate levels, which were directly measured form the tail vein before and after the exercise (Fig. 5B). This is what is expected if the level of blood lactate is a main reason for why the animals stop running. No significant difference between genotypes was observed. We interpret these findings to indicate that animals of both genotypes stop running at the same level of blood lactate but that *Mclk1*^*+/−*^ mice sustain a more rapid buildup of lactate levels. Thus, it appears that insufficient aerobic capacity is likely the cause of the low exercise capacity in *Mclk1*^+/−^ mutants. Analysis of blood glucose concentration revealed a trend for an increase after the treadmill exercise which did not differ between genotypes (Fig. 5C).

### Enhanced voluntary activity in RISP^+/P224S^ mitochondrial mutants

**Figure 6.**
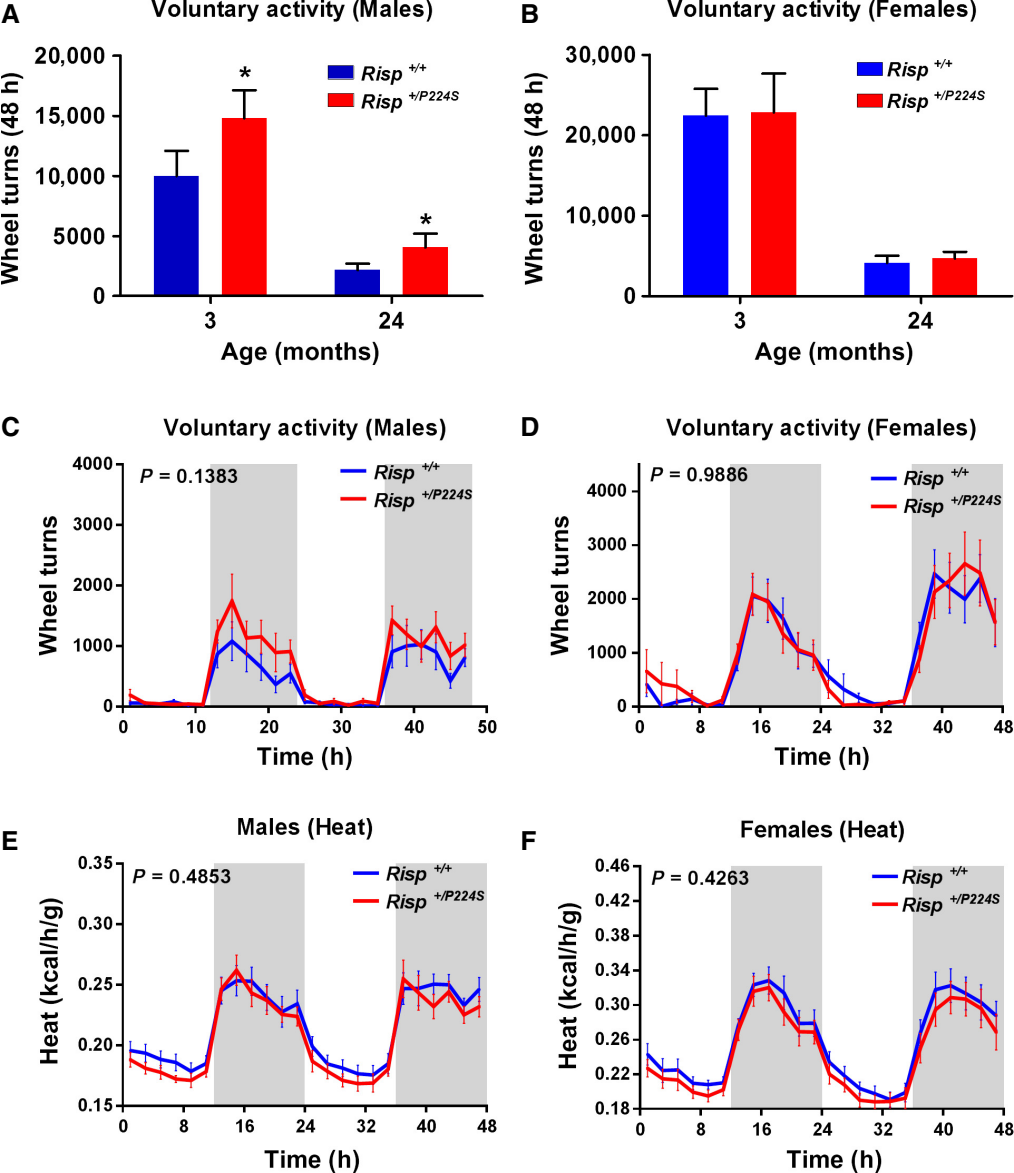
Increased voluntary activity and metabolic rate in *RISP*^+/*P224S*^ males in presence of a running wheel. Total number of wheel turns accomplished by 3‐ and 24‐month‐old *RISP*^+/*+*^ and *RISP*^+/*P224S*^ mice (*n* = 15–20) during the entire 48 h period: males (A) and females (B). Bars represent means ± SEM and a value of *P* < 0.05 was considered significant. Voluntary activity on running wheel recorded at 2 h intervals throughout the 48 h period with a 12 h light and 12 h dark cycle for both sexes at 3 months of age (C–D). Heat production was also assessed by indirect calorimetry for the entire experimental period (E–F). The shaded areas demarcate the dark phases (from 7:00 pm to 7:00 am). Time “0” is 7:00 am. All points represent means ± SEM over 2 h intervals. A value of *P* < 0.05 for the genotype effect was considered significant.

As in *Mclk1*^+/−^ mice, mitochondrial electron transport is impaired in *RISP*^+/*P224S*^ mice and this leads to a significant decreased whole‐body metabolic rate for the males (Hughes and Hekimi 2011). We sought to determine whether this also led to a change in spontaneous activity as in *Mclk1*^+/−^ males. In addition, we scored the influence of age and sex on voluntary activity by using 3‐ and 24‐month‐old males and females, which were all evaluated over a 48 h period. We observed that the *RISP*^+/*P224S*^ males were significantly more active at both 3 and 24 months of age, but females were unaffected (Fig. 6A–D). Furthermore, the metabolic rate (heat production) of the *RISP*^+/*P224S*^ males, which was found to be lower than normal without running wheels (Hughes and Hekimi 2011), is restored to control values when they could use a running wheel (Fig. 6E). The level of heat production was not affected by the availability of a running wheel in 3‐month‐old *RISP*^+/*P224S*^ females (Fig. 6F), consistent with their unchanged activity level relative to wild‐type controls (Fig. 6F). We further observed that the total number of wheel turns is higher for *RISP*^+/*+*^ than for *Mclk1*^*+/+*^ 3‐month‐old controls (Fig. 6A–B; compare to Figs. 2A, 3A), a difference that is, likely the result of different genetic backgrounds, as has been previously documented (de Visser et al. 2007).

## Discussion

### Mitochondrial dysfunction affects whole‐body energy metabolism in Mclk1^+/−^ males but not females

The long‐lived *Mclk1*^+/−^ mutants are characterized by an abnormal distribution of UQ in mitochondrial membranes which is linked to decreased oxygen consumption, electron transport rate, and ATP production measured in isolated mitochondria from several tissues including liver, kidneys, heart, and skeletal muscle (Lapointe and Hekimi 2008; Lapointe et al. 2012). These results were obtained with purified mitochondria in vitro, which provide information about the maximal mitochondrial capacities. Here, we show that whole‐body energy metabolism is also altered in 3‐month‐old male mutants but not in females, despite the fact that their mitochondria were equally affected in vitro (Figs. 1, 2). For *RISP*^+/*P224S*^ mice we also reported sex‐specific effects (Hughes and Hekimi 2011). However, in this case both ETC function of purified mitochondria and whole‐animal metabolism were only affected in males. A preferential sensitivity of males was also found in other models of mitochondrial dysfunction (Diaz et al. 2005; Yang et al. 2010). The cause of any of these sex‐specific effects is still unknown.

Interestingly, rat mitochondrial oxidative metabolism is sexually dimorphic, with isolated mitochondria from several female organs having greater respiratory capacities as revealed by morphologic differences (greater mitochondrial size and higher cristae density) and more active mitochondria in respiratory state 3 (Justo et al. 2005). This greater mitochondrial oxygen consumption in females was associated with higher UCP1 content (Rodriguez‐Cuenca et al. 2002). UCP1 is an inner‐membrane mitochondrial protein that uncouples the respiratory chain from ATP synthesis by dissipating the proton gradient generated by the respiratory chain as heat (Ricquier and Bouillaud 2000). Thus, female rats are more thermogenic than males. If this is also true for female mice, this might help to explain why the resting metabolic state of *Mclk1*^+/−^ females did not reflect their mitochondrial ETC dysfunction (see below).

### Increased voluntary activity in mitochondrial ETC mutants

Our study sought to investigate the metabolic, physiological, and behavioral adjustments that accompany altered mitochondrial function in *Mclk1*^+/−^ and *RISP*^+/*P224S*^ mice. Running wheels are commonly employed to measure rodent physical activity in a variety of contexts (Richter et al. 2008; Novak et al. 2012). The use of running wheels modifies several aspects of energy balance by increasing activity and energy expenditure in rodents. Wheel‐running behavior appears to be a complex and dynamic behavior where genetics and the environment interact (Joosen et al. 2005; Garland et al. 2010; Kelly et al. 2012). Enhanced voluntary wheel running has been observed for mice with a knockout of the G protein‐coupled receptor GPRC6A which has been hypothesized to regulate exercise behavior (Clemmensen et al. 2013) as well as in animals with a deletion of the glucose transporter GLUT8 (Schmidt et al. 2008). Here, we found that two independent mutant mice strains characterized by impaired mitochondrial ETC function and decreased whole‐body energy metabolism had increased running wheel activity. Analysis of metabolic parameters of both *Mclk1*^*+/−*^ and *RISP*^+/*P224S*^ mutants revealed that this increase in activity was sufficient to alleviate the depressed oxygen consumption and heat production levels observed in these mutants at rest (Figs. 2, 6). Despite increased levels of activity, metabolic rate was not significantly increased above their less active wild‐type controls. It is believed that one of the important parameters that determine metabolic rate is the need to maintain an adequate and constant body temperature (Rolfe and Brown 1997; Rhodes et al. 2000). Thus, the purpose of the increased locomotory activity in the mutants might in fact be a need to increase thermogenesis.

In *Mclk1* mutants, both males and females have impaired mitochondrial function as established by in vitro studies (Lapointe and Hekimi 2008) and both show increased voluntary running wheel activity (Figs. 2, 3). However, only males and not females display a low VO_2_ (Fig. 1). One possibility to explain this discrepancy is that females possess mechanisms of thermogenesis that males do not possess and that do not require increased locomotory activity. The observation that UCP1 uncoupling protein is highly expressed in brown adipose tissue of female rats suggests that regulation of thermogenesis is sex‐specific in rodents (Valle et al. 2007). Thus, in the absence of running wheels, female‐mutant VO_2_ is higher than in male mutant in spite of similar mitochondrial defects because oxygen consumption is stimulated by a female‐specific mechanism of thermogenesis. Males on the other hand need the running wheels to reach the same normalization of VO_2_. It is unclear why female mutants still have increased locomotory activity. There might be other benefits of increased locomotory activity for animals with defective mitochondria besides thermogenesis or increased VO_2_. Alternatively, this behavior might be maladaptive but further experiments are required to clarify these results.

### Increase mitochondrial oxidative stress is not stimulating voluntary wheel running

As mentioned earlier, the ETC defects of *Mclk1*^+/−^ mice are accompanied by increased mitochondrial oxidative stress (Lapointe and Hekimi 2008). To better understand the relation between mitochondrial oxidative stress and voluntary activity we studied *Sod2*^+/−^ and *Sod2*^+/−^ *Mclk1*^+/−^ double mutant mice. We found that 15‐month‐old *Sod2*^+/−^ mutants, which are known to sustain high mitochondrial oxidative damage (Williams et al. 1998; Melov et al. 1999; Van Remmen et al. 2003), were not very active on the running wheel (although the difference with wild‐type siblings was not statistically significant). Similarly, no differences in metabolic rate and voluntary activity were observed between *Mclk1*^+/−^ and *Mclk1*^+/+^ control mice at 15 months of age (Fig. 4). While this contrasts with our findings with 3‐month‐old mice, it is consistent with many previous observations showing that the phenotypes of *Mclk1*^+/−^ mice revert to the wild type with age (Lapointe et al. 2009). Furthermore, loss of one copy of *Mclk1* in the 15‐month‐old *Sod2*^+/−^ *Mclk1*^+/−^ double mutants significantly enhanced voluntary activity in comparison to *Sod2*^+/−^ mutants. This is consistent with previous observations showing that *Mclk1* heterozygosity has a marked impact on *Sod2*^+/−^ phenotypes, in particular mitochondrial oxidative stress (Lapointe et al. 2009). The mechanism of this suppression remains unexplained. Interestingly, it was also shown that the absence of the major cytoplasmic superoxide dismutase in *Sod1*^−/−^ mice leads to an increase in the levels of oxidative damage in skeletal muscle which is accompanied by a significant decrease in voluntary wheel running and physical performance (Muller et al. 2006). Together these results strongly suggest that mitochondrial oxidative stress is not the factor responsible for triggering increased voluntary activity in *Mclk1*^+/−^ mice.

### Mitochondrial oxidative stress negatively impact physical performance

The study of the key factors differentiating the running and nonrunning strains in mice strongly suggest that wheel‐running motivation is not necessarily linked to performance capacity (Rezende et al. 2005). The highly active *Mclk1*^+/−^ mice were thus subjected to an endurance treadmill test in order to evaluate their physical performance. We found that exercise capacity is compromise in these animals as revealed by decreased distance run before exhaustion (Fig. 5). Furthermore, blood lactate levels in mutant and control mice were similar at the time of exhaustion, although mutant mice stopped running much earlier. Based on these observations, it is reasonable to speculate that lactate accumulates more rapidly in the blood of the mutants during forced exercise. There is a growing body of literature indicating that mitochondrial ROS are a major cause of muscle fatigue (Reid 2008; Westerblad and Allen 2011). Accordingly, similar treadmill tests have revealed that exercise capacity is limited in *Sod2*^+/−^ mice (Kinugawa 2005), as well as in skeletal muscle‐specific *Sod2* deficient mice (muscle‐*Sod2*^−/−^; Kuwahara et al. 2010). The muscle‐*Sod2*^−/−^ mice show severe exercise weakness accompanied by a mitochondrial phenotype which highly resembles that of *Mclk1*^+/−^ mice, with a significant decrease in enzymatic activity for mitochondrial respiratory chain complexes and reduced ATP synthesis. Furthermore, as in *Mclk1*^+/−^ mutants (Lapointe and Hekimi 2008), the reduced muscle ATP content in muscle‐*Sod2*^−/−^ mice does not affect skeletal muscle function during nonforced tests such as spontaneous activity. A rescue experiment using the superoxide dismutase mimetic EUK‐8 has resulted in a significantly improvement in exercise capacity, increased cellular ATP content and reduced blood lactate levels in muscle‐*Sod2*^−/−^ mice (Kuwahara et al. 2010).

Another *Sod2* conditional knockout has been produced by use of *Sod2*^*fl/fl*^ with a *Cre* recombinase driven by the promoter for the inhibitory subunit of troponin (*TnIFastCre*; Lustgarten et al. 2009). The resulting mice were also found to have increased mitochondrial oxidative damage in glycolytic muscles and to run a significantly lesser distance on a treadmill than controls. Blood glucose levels were not significantly different between these mutants and wild‐type mice after running but a greater rate of glucose utilization and increased blood lactate levels were measured from the onset of running until exhaustion, which is reached more rapidly in mutants. Similarly, it was shown that treating mice with angiotensin II decreases mitochondrial complex I and II activities, induces superoxide production and seriously impairs exercise capacity (Inoue et al. 2012). Moreover, a recent study has revealed that dietary supplementation with specific molecules known for their antioxidant properties such as coenzyme Q, vitamin E, and *α*‐lipoic acid improves mitochondrial function and augments running performance in untrained mice (Abadi et al. 2013). Interestingly, these effects were only observed in female. On the other hand, it was also proposed that dietary supplementation with antioxidants may preclude the long‐term health‐promoting and adaptive effects of exercise‐induced oxidative stress (Ristow et al., 2009). Collectively, our findings and the various studies mentioned suggest that elevated mitochondrial oxidative stress is sufficient to reduce exercise capacity.

## Conclusion

In summary, the results presented here shed light on the biological links between mitochondrial function, whole‐body metabolic rate, voluntary activity, and exercise capacity. By using different genetically engineered mice with specific mitochondrial dysfunction phenotypes such as the long‐lived *Mclk1*^+/−^ mice, we showed that defects in mitochondrial respiratory chain lead to a sex‐specific decrease in metabolic rate and increased voluntary use of a running wheel. However, this increased voluntary activity was not reflected in exercise capacity, which was impaired in *Mclk1*^*+/−*^ mice.

## Acknowledgment

We thank Z. Stepanyan for preliminary results on body weight and food intake in *Mclk1*^+/−^ mice.

## Conflict of Interests

None declared.
